# My Diet Study: protocol for a two-part observational, longitudinal, psycho-biological study of dieting in Australian youth

**DOI:** 10.3389/fpubh.2023.1281855

**Published:** 2023-12-14

**Authors:** Mirei Okada, Melissa J. Pehlivan, Jane Miskovic-Wheatley, Sarah Barakat, Kristi R. Griffiths, Stephen W. Touyz, Stephen J. Simpson, Sarah Maguire, Andrew J. Holmes

**Affiliations:** ^1^Charles Perkins Centre, The University of Sydney, Camperdown, NSW, Australia; ^2^School of Life and Environmental Sciences, The University of Sydney, Camperdown, NSW, Australia; ^3^InsideOut Institute for Eating Disorders, The University of Sydney and Sydney Local Health District, Camperdown, NSW, Australia

**Keywords:** self-directed dieting, feeding behavior, gut-brain axis, metabolomics, microbiome, youth, protocol

## Abstract

**Introduction:**

Self-directed dieting (i.e., unsupervised) is very common among adolescents and young adults but has had almost no direct research. This paper describes the protocol for the My Diet Study, a two-arm observational investigation of the natural progression of dieting among young people over a period of 6-months. The study aims to examine the links between self-directed dieting, general physiological and psychological metrics of wellbeing (e.g., depressive symptoms) and biomarkers of gut-brain axis functions (e.g., microbiome and hormones) that are predicted to influence diet adherence through appetite, mood and metabolism regulation.

**Methods:**

Young people aged 16–25, intending to start a diet will be invited to participate in this observational study. For Part 1 (psychological arm), participants will be asked to complete a set of questionnaires and diaries at the beginning of every month for 6 months, to assess overall mental (e.g., psychological distress, disordered eating) and physical (e.g., weight) health, perceived diet success, food intake and gastrointestinal movements. For Part 2 (biological arm), a subsample of 50 participants will be asked to provide feces, blood and saliva for bio-sampling each month for the first 3-months of their participation in Part 1.

**Discussion:**

The My Diet Study will be the first longitudinal, observational study of dieting in young people combining in-depth psychological and biological data. It is anticipated that the findings will yield psychological & biological information about the impacts and effectiveness of self-directed dieting in young people, inform a framework for advice on safety in dieting among young people and help to establish the potential for biomarkers for risk management and improvement of diet-based lifestyle interventions.

## 1 Introduction

Our diet (i.e., the pattern of intake of food components for an individual integrated over time) (1–4) has wide ranging impacts on our health, mood (5) and appearance (6–9). In addition, our food choices as a society impact the broader environment (10). There are many reasons why individuals may choose to deliberately alter their eating pattern (i.e., dieting or “going on a *diet*”), some of these reasons may be healthy, whilst other are not and include multiple influencing factors (e.g., nutrition, intolerances, values, aesthetics, cultural reasons, alteration in the timing of food intake). These dieting have complex relationship with risks and health benefits to (8) self-directed dieting which is common in young people. For example, European and USA studies have estimated the prevalence of dieting over 12-month windows to be up to 64% of young females and 44% of young males [age range: 17–32 yrs; (11–13)]. Despite this, we have a limited understanding of the relationship between motivating factors, self-directed diet planning, and outcomes.

By our definition “*dieting*” is change, both nutritionally and as a chosen behavior. Behavior change theories (14, 15) identify three main phases that determine successful change: motivation, planning and action. This paper describes a protocol to explore the interaction between these behavioral aspects of dieting with the psycho-physiological consequences of altered eating patterns. When “dieting”, an individual self-regulates their food intake by following a set of guidelines or rules that shape one or more of these aspects of food intake (16). Nutritionally, this may include the amounts of macronutrients (e.g., protein, carbohydrate, fats, fiber) or energy (e.g., caloric restriction diets). If continued in the long-term (e.g., 6+ months), it may become part of a new eating pattern [now referred to as their diet, or habitual food intake (17)]. However, most people have difficulty adhering to diets even in the short-term (18, 19), particularly weight-loss diets (20, 21), which are often unsuccessful over the long-term [e.g., >1 year (22)]. We postulate this reflects interaction between physiological and psychological drives of eating behavior.

Generally, dieting motivations lie across three broad categories: health (e.g., to improve cardiovascular health, to avoid inflammation), appearance (e.g., body composition, complexion) and value-based reasons (e.g., animal welfare or environmental concerns, cultural or religious reasons) (8, 20, 23). The motivation to undergo diet change also likely influences how the diet is planned (e.g., formation of diet rules) and whether the planned diet is feasible and likely to deliver the expected outcomes will also impact compliance and outcomes. Self-directed dieters may not be aware of the different satiating properties of various macronutrients and not include satiating foods (e.g., high protein or high fiber foods) which help with adherence (24, 25) and may improve outcomes [e.g., weight-loss, body composition (26–28)]. Unguided in their diet journey, self-directed dieters may also take a rigid approach (e.g., complete abstinence from forbidden foods) toward dietary restriction, which has been linked with greater food cravings (29), overeating (30), weight regain (31) and diet abandonment (32).

While dieting is a chosen behavior, driven partly by top-down processes [e.g., self-control (33)], there are many unconscious influences which may also determine compliance and outcomes. For example, environmental factors have been found to unconsciously influence eating behaviors, including the presence of others while eating (34, 35), portion sizes and food packaging (36, 37). We also know there are numerous physiological factors, including appetite hormones, the gastrointestinal tract, circulating metabolites and nutrients, organoleptic compounds, toxins, and the immune system, which work together via the brain to subconsciously determine food intake (38–40). There are two primary biological systems thought to be involved in feeding behavior: the homeostatic system (i.e., appetite hormones, hypothalamic pathway) which works to maintain an appropriate energy balance; and the hedonic system (i.e., brain reward centers and pathways) which seeks out and pursues reward (e.g., palatable food) (41). These two systems work together to influence feeding behavior (42, 43). Whether a diet is maintained likely depends on the physical and mental health consequences and rewards of following the diet (44). These consequences may be intentional (i.e., aligned with diet motivation) or unintentional, and positive (e.g., increased sense of mastery or control, reducing cholesterol) or negative (e.g., feelings of deprivation, low mood, bloating) (45).

**Figure 1:**
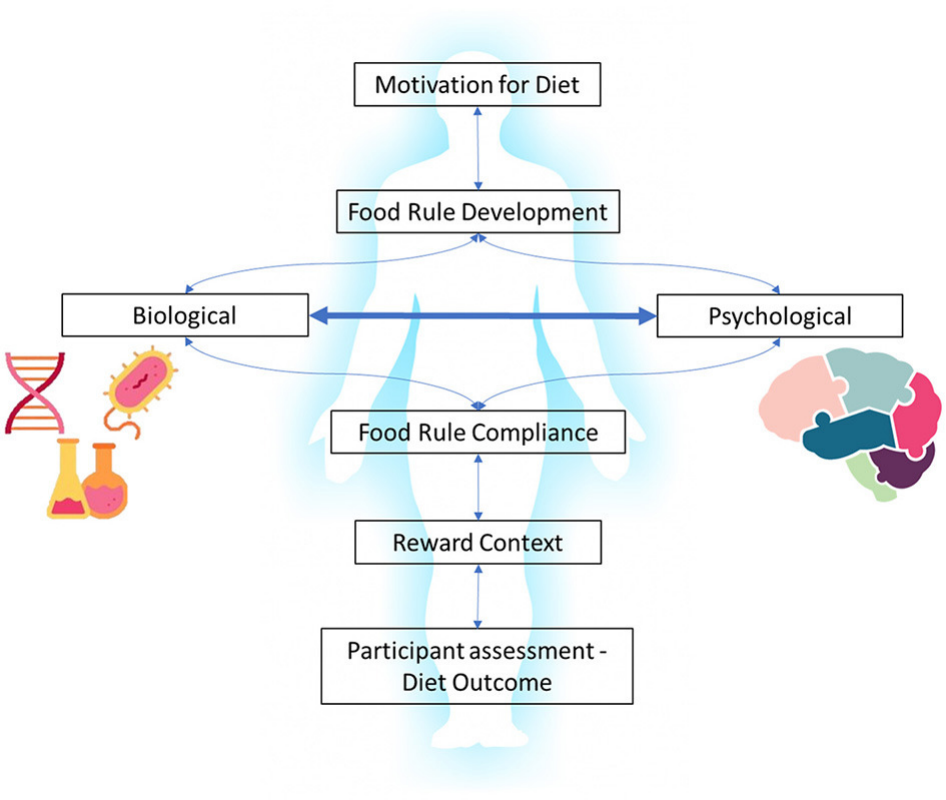
Two main outcome domains of dieting.

How these “rewards” and “consequences” interact to drive both diet compliance and diet outcome is a fundamental knowledge gap. There are two different outcome domains in the dieting process: psychological and biological. They are inter-related and at the same time asymmetrical in that a positively experienced outcome at a psychological level can be a negative physical health outcome and vice versa. These are the properties of a complex adaptive system, in which feedback occurs across multiple domains, at multiple levels, and single outcomes can have different impacts on different parts of the system (Figure 1).

Some evidence suggests diets can have positive effects, at least in the short term, for a wide range of clinical conditions including immuno-metabolic [e.g., diabetes, obesity, irritable bowel syndrome (26, 46)], and some psychiatric symptoms [e.g., reduced depressive symptoms, (45, 47, 48)]. However, these positive effects have only been found with supervised diets, which are set and monitored by a qualified health professional or consumer support organization (49). The longer-term risks associated with dieting, in particular in young people, are poorly understood. It is possible that dieting for appearance-based reasons may be disproportionately represented in young people (50, 51). Past research has indicated that weight-loss dieting may be the single strongest predictor of new cases of an eating disorder (52). Research is needed to clarify the role of dieting as a risk factor for an eating disorder vs. dieting as an “early sign” of the onset of an eating disorder.

The My Diet Study is an observational, longitudinal 6-month study of unsupervised dieting in the very population who frequently engages in this behavior—young people. The first in-depth study of its' kind, the My Diet Study is an exploratory study into the psychological and biological influences that drive diet compliance and outcomes. We aim to examine the impact and interplay between psychological and physiological response mechanisms (e.g., gut microbiome, appetite-hormone) which may feedback to influence food intake behaviors, adherence, diet success and outcomes (physical and mental health, e.g., weight, depression, disordered eating).

## 2 Methods and analysis

### 2.1 Study design

**Figure 2:**
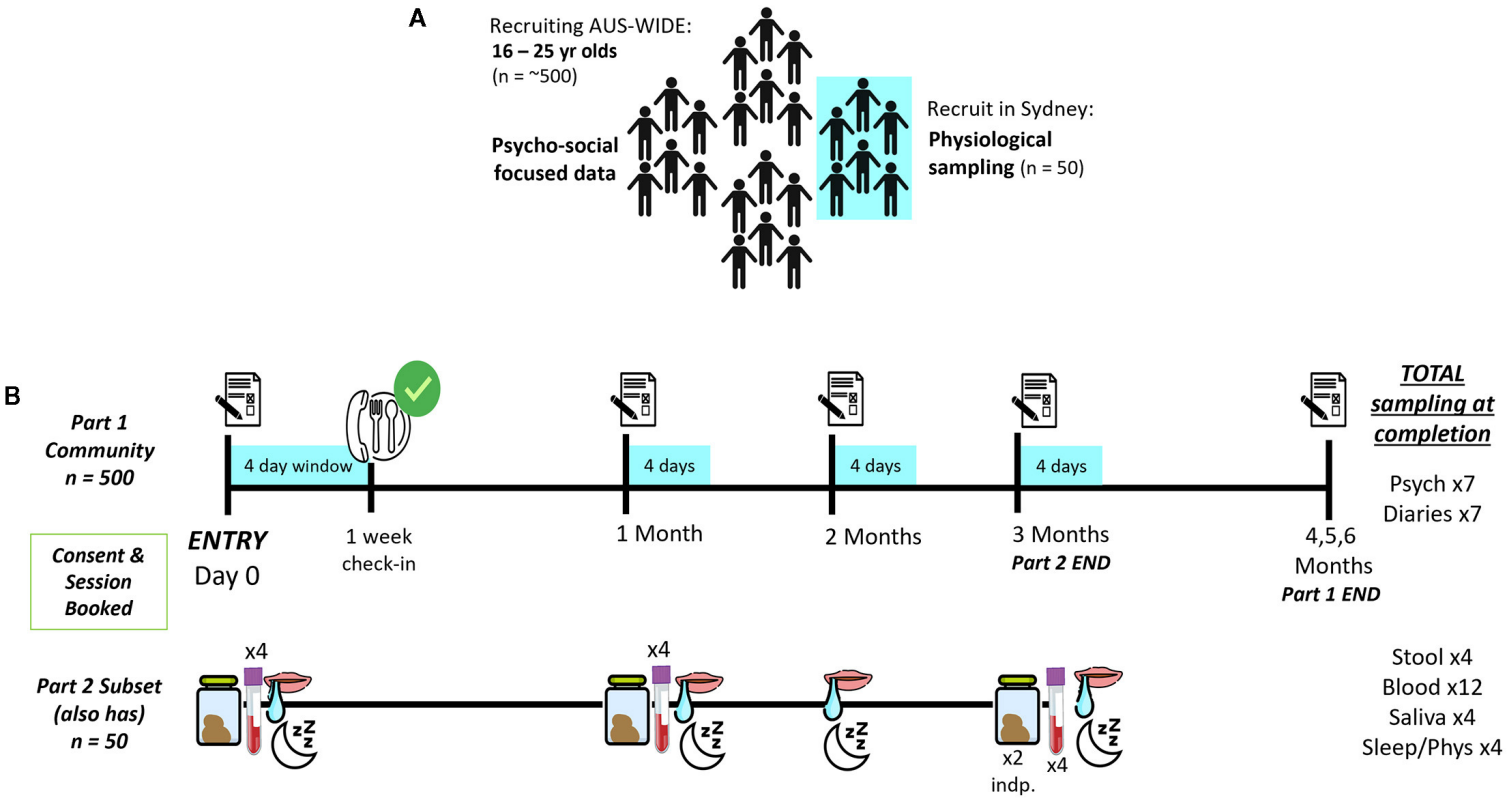
Design and assessment schedule of concurrent two-part study.

This is a two-part observational and longitudinal study in Australian youth (see Figures 2A, B). Part 1 consists of a 6-month series of monthly online questionnaires and 4-day diaries to assess self-reported psychological (e.g., motivation, perceived success) and physical (e.g., satiety, gastrointestinal movements) aspects of unsupervised dieting in a representative sample (*N* = 500) of young people (aged 16–25 years) (Figure 2B). The 6-month time period was chosen as evidence suggests behavioral eating habits form anywhere between 18–254 days (17). In Part 2, a subset (*n* = 50) of Part 1 will provide biological samples (e.g., stool/feces, blood, saliva) each month for the first 3-months of their participation in Part 1, to identify physiological factors which may impact food intake, diet success and outcomes (Figure 2B). This protocol was developed in collaboration with experts in microbiology, research processes, mental health and eating disorder experts, including consultation with those with a lived experience of an eating disorder. Further, the protocol was approved by the Human Ethics Review Committee (RPAH Zone) of the Sydney Local Health District (X21-0181). The study will be conducted in accordance with the *National Statement on Ethical Conduct in Human Research* (2007), the *CPMP/ICH Note for Guidance on Good Clinical Practice* and consistent with the principles that have their origin in the Declaration of Helsinki.

### 2.2 Participants

**Figure 3:**
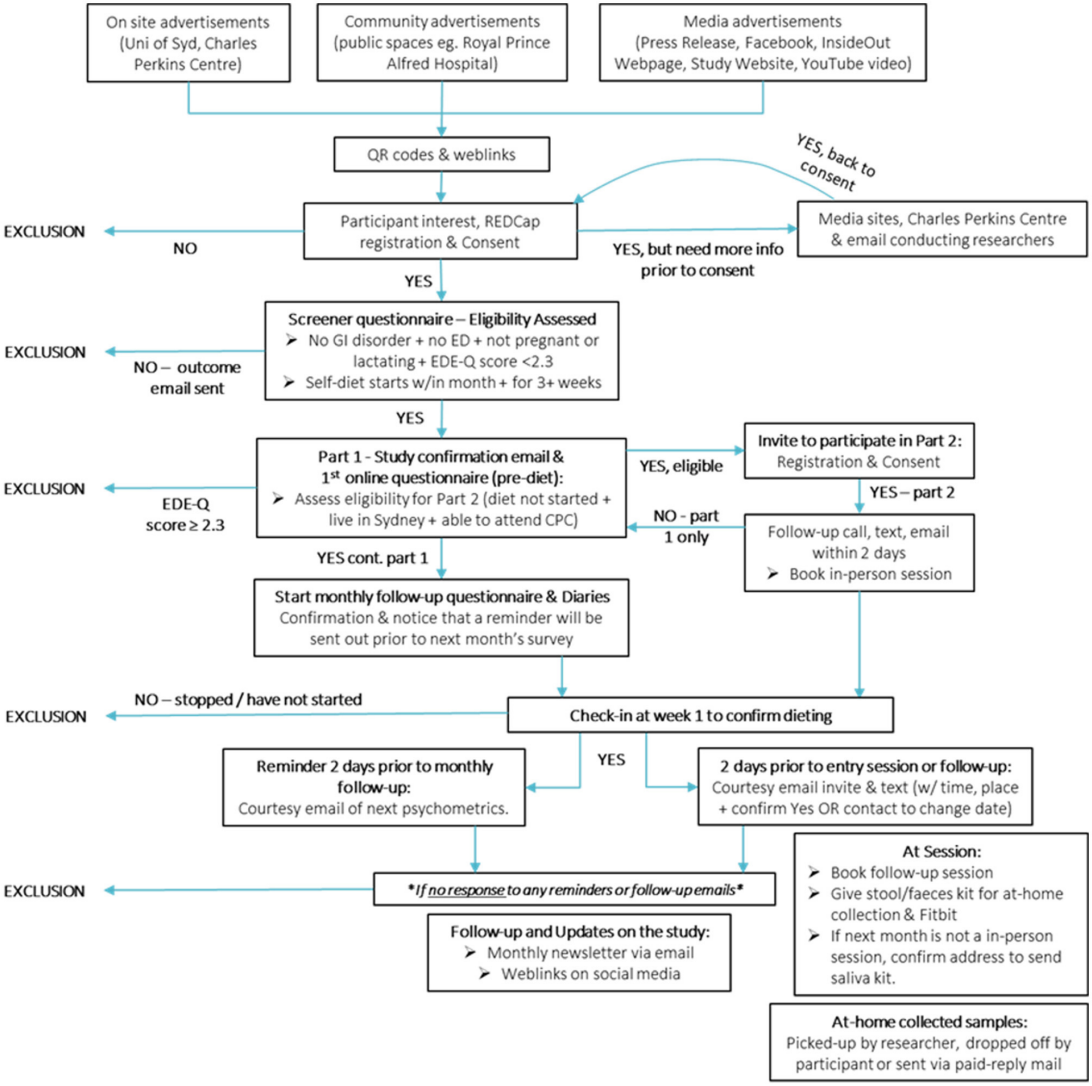
Participant entry into parts 1 and 2.

Potential participants will be recruited from the general community using advertisements online and at local universities and TAFE campuses and clinics (see Figure 3). Social media (e.g., Facebook, Instagram, Reddit), as well as the InsideOut Institute and designated study website^1^ will be used to recruit a geographically diverse sample. To promote retention for the duration of the 6-month study period, participants will be reimbursed (online gift cards) for their time and there will be additional incentives for Part 2 (Fitbit, gut microbiome profiling report, additional online gift cards). Local students enrolled in psychology classes at the University of Sydney will also be invited to participate in return for partial course credit. Eligibility criteria for Part 1 are listed in Table 1.

**Table 1:** Study inclusion and exclusion criteria.

**Inclusion criteria**	**Exclusion criteria**
Parts 1 and 2 •Aged 16–25 years old (inclusive) •Starting a diet lasting longer than 2 weeks •BMI ≥ 17.5^*^ •English language speaking/reading •Internet access and computer literacy Part 2 only•Able to attend the University of Sydney's Charles Perkins Centre •Intention of dieting for at least 3 months	Parts 1 and 2 •EDE-Q global score ≥ 2.3 (at-risk cut-off) •Diet prescribed by medical practitioner (i.e., supervised diet) •BMI <17.5^*^ •Current or past eating disorder^*^ •Formal diagnosis of malnourishment^*^ •Pregnant or lactating^*^ Part 2 only•Started a self-directed diet already •Treatment of antibiotics or steroids in the last month •Major medical condition^*^

^*^Indicated self-reported.

Participants will view a detailed description of the study online prior to giving online informed consent. After giving online consent, participants will complete a brief online screener questionnaire via University of Sydney's licensed online data collection portal, Research Electronic Data Capture [REDCap (53, 54)], a secure online program for developing and administering online surveys that is compliant with standard privacy and security requirements (e.g., HIPPA, FISMA). Eligible participants will be asked to complete the baseline survey and start logging their 4-day (3 week day, 1 weekend day) daily diaries the day they start their diet, via Teamscope, an online data collection application, which participants can download using iOS and Andriod phones. Teamscope enables frequent and rapid data collection, such as online diaries, having an easy to use interface and being able to collect data offline (i.e., without internet connection). Further, Teamscope is fully compliant with HIPAA, GDPR and Good Clinical Practice (ICH-GCP).

Eligible participants for Part 2 will be notified and invited to provide biological samples. Only individuals from Greater Sydney will be invited to ensure feasibility of participants attending multiple appointments. Consenting participants for Part 2, will be asked to have fasted for 6–8 h overnight prior to coming into the Charles Perkins Centre, University of Sydney for their in-person biological sampling sessions. Participants will be asked to bring in their breakfast to be consumed after their first blood draw.

**Figure 4:**
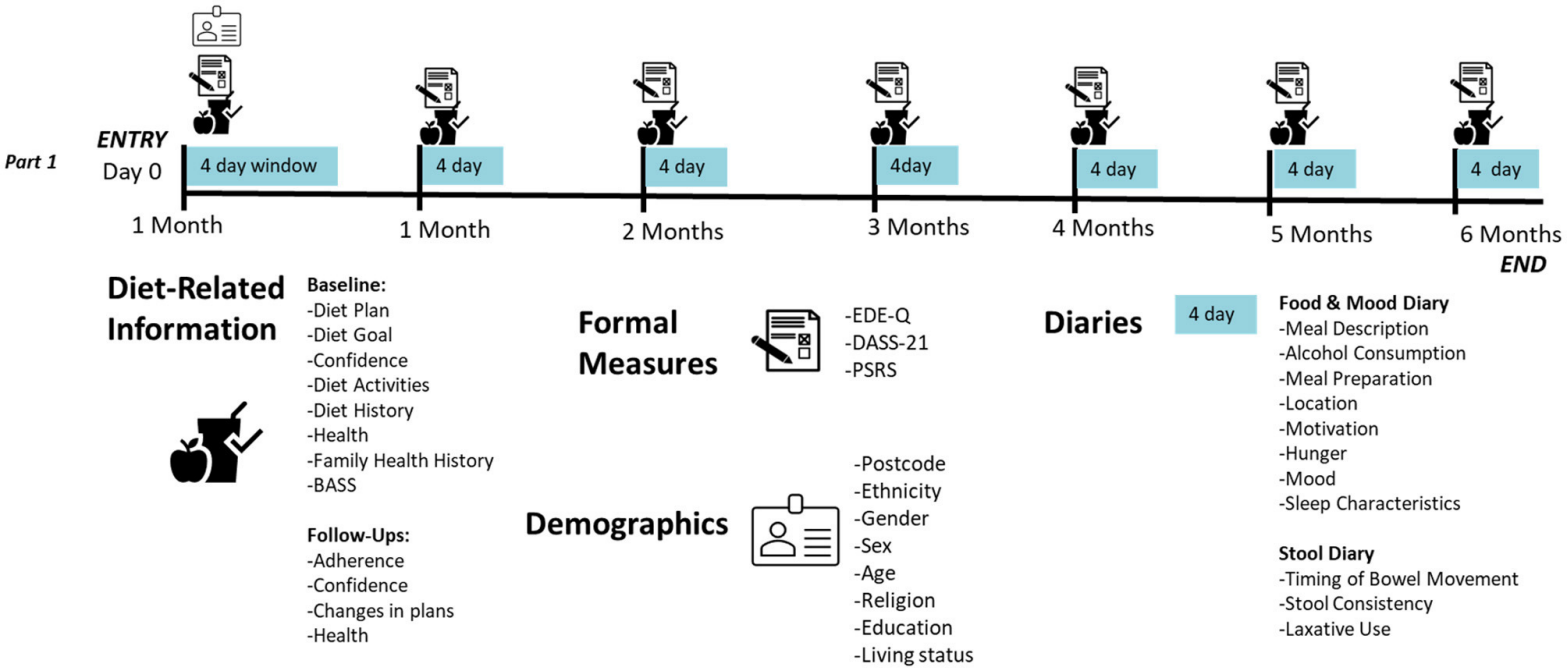
Detailed overview of part 1 assessment schedule.

Each month, participants will receive an email with a personalized link to complete their monthly follow-up questionnaire (see Figure 4 for an overview of Part 1 measures) on REDCap, and a reminder to complete their 4-day daily diaries for the month. For ethical and safety reasons, participants will be monitored for changes in physical and mental health by clinicians. Any participant meeting full threshold DSM-V (55) criteria for an ED (determined by responses on the Eating Disorder Examination Questionnaire (EDE-Q) collected monthly (56) and a phone consultation with a psychologist—SB, SM, JMW, ST) or indicates medical [e.g., rapid weight loss, low Body Mass Index (BMI), new medical condition] or psychological/behavioral (e.g., purging behaviors, frequent binge eating episodes) risk will be withdrawn by the study clinician (SB) and referred on to appropriate support services.

Should participants miss two or more consecutive monthly follow-ups, they will be thanked for their participation and withdrawn from future participation in the study. Withdrawn participants may keep all incentives up until the point of exclusion. Participants who have decided to pause or end their diet, will be encouraged to continue completing study measures and receive associated incentives, as we are interested in their dieting journey. Only participants who complete all measures will receive study incentives.

### 2.3 Measures

#### 2.3.1 Sociodemographic and medical factors

At baseline, sociodemographic (e.g., gender, ethnicity, religion, education, location, employment and living status) and health information (e.g., underlying health conditions, mental health diagnoses, family history of eating disorders and obesity) will be collected using a mixture of multiple-choice and open-ended questions (see Supplementary material 1). Each month, participants will be asked to report on their physical health, identifying any new conditions and treatment with antibiotics or steroids. Further, at baseline and each month, participants will report on their diet, including their diet history, motivation, goals, confidence and plans through a combination of open-ended, multiple choice and sliding scale questions (see Supplementary material 1).

##### 2.3.1.1 Part 1 (psychological arm): formal measures

Participants' psychological distress (depression, stress, anxiety) will be assessed with the widely used short (21-item) self-report Depression Anxiety Stress Scales [DASS-21 (57, 58)]. The DASS-21 demonstrates discriminant validity (57) and sensitivity to change (59), and thus should be helpful in tracking participants' psychological wellbeing over time. Further, the DASS-21 was developed and validated among Australian undergraduate students (57).

Participants' eating disorder psychopathology will be examined using the EDE-Q (60, 61), a 28-item self-report measure, derived from the gold standard clinical interview, the Eating Disorder Examination [EDE (62)]. Unlike the EDE, the EDE-Q is highly time and cost effective to administer (taking ~15 min to complete and not requiring clinician administration), making it ideal for use in large community samples (60). Using the same probing questions as the EDE, the EDE-Q assesses participants' attitudes and behaviors related to food, body weight and shape over the past 28 days (60). In this study, the conservative clinical cut-off of a global score of at least 2.3 (63), will be used to identify individuals at risk of an ED for exclusion prior to study entry. The EDE-Q has demonstrated good discriminative validity, differentiating between those with and without an eating disorder (63–66).

Participants' body dissatisfaction will be assessed at baseline using the Body Areas Satisfaction Scale [BASS (67)], a 9-item subscale of the Multi-dimensional Body Self-Relations Questionnaire [MBSRQ (68)]. Appearance concerns are a common reason for dieting (69), particularly among adolescents (23). Intended for use with adults and adolescents, the BASS has demonstrated good internal validity [α = 0.73–0.77; (67)].

Participants' weight loss efforts will be assessed using the 3-item Perceived Self-Regulatory Success in Dieting Scale [PSRS (70)]. Measures of dietary restraint [e.g., Dutch Eating Behavior Questionnaire, (71), Restraint Scale (RS), (72)] are not able to identify those dieters who attain a calorie deficit (73) and some (e.g., RS) have been found to measure dieters who are more inclined to gain weight (74). The PSRS has demonstrated good convergent validity, being negatively associated with BMI and binge eating frequency (73).

Participants will be asked to log each meal for the first 4 days of the month in response to a series of open-ended and multiple-choice questions (see Supplementary material 2). Participants responses will be examined for adherence to their planned diet, as well as influencing factors on their food intake (e.g., physical and social setting, mood, hunger and satiety). Sleep information will also be ascertained during the first meal log of the day, given its influence on food intake (75). The daily diaries also include multiple choice questions to collect gastrointestinal movements over the preceding period (see Supplementary material 2). In their responses, participants will use the Bristol Stool Chart (76), a diagnostic medical tool, to classify their fecal matter. For participants in Part 2, a prompt is also raised to indicate which sample is being used for deoxyribonucleic acid (DNA) extraction and microbiome analysis.

##### 2.3.1.2 Part 2 (biological arm): formal measures

To characterize the impact of longitudinal dieting, a cohort of 50 participants from Part 1 will have their response in physiology and observed standard metrics assessed (microbiome, endocrine, weight/fat). Morphometric, biological sampling and Fitbit physiology tracking (sleep and heart rate) from participant visits at the in-patient facility at the Charles Perkins Centre-Royal Prince Alfred clinic will be supplemented by the daily diaries.

**Figure 5:**
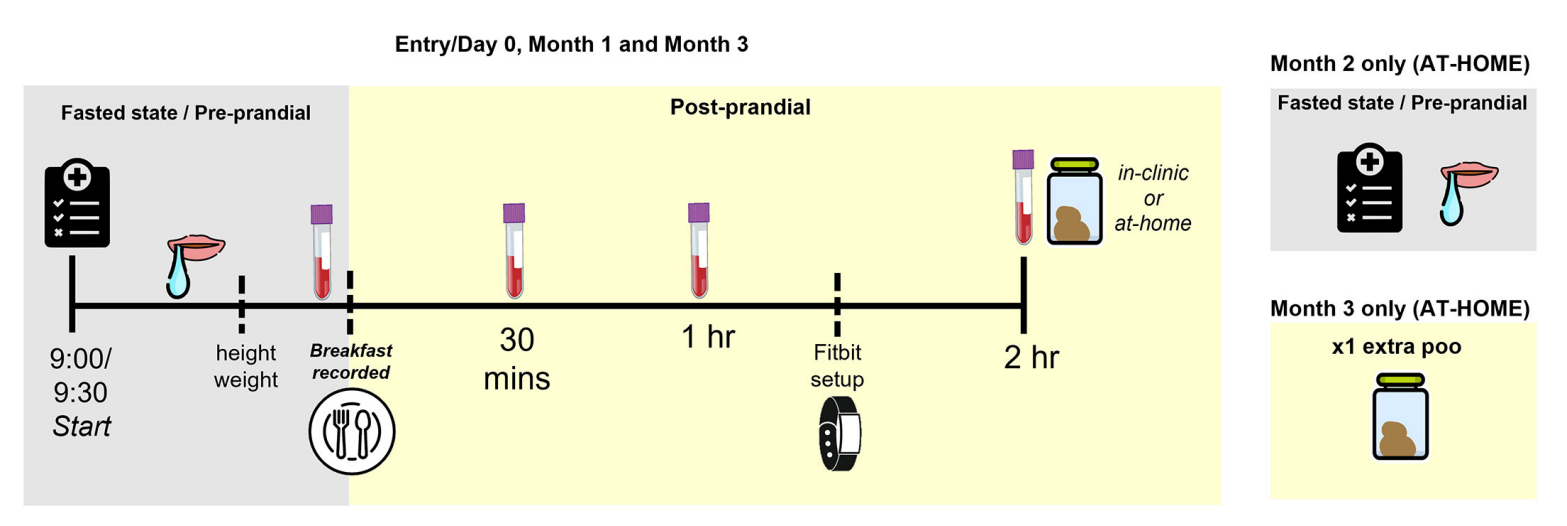
Overview of part 2 in-clinic assessments.

At each in-person session the following data will be collected (see Figure 5 for more detail):

- Standard health questions pre-sample collection (e.g., “Did you fast prior to coming and only had water?”, “Have you fainted before during a blood collection?”, “Are you allergic to any medical tapes, isopropyl alcohol or latex?”)

° Includes NSW Health Sydney Local Health District required COVID-19 screening night prior to in-clinic assessment

- Morphometrics—weight, height and BMI will be determined at each participant visit. These data will be supplemented with participant self-reported weight measures indicating individuals weight fluctuations.
- Blood pressure, heart rate and oxygen saturation—using an automated blood pressure monitor.
- Circulating biomolecules (appetite hormones, cytokines and metabolites)—from blood plasma collected in vacutainers (Becton Dickinson) pre-coated with K_2_EDTA and cocktail of protease, esterase, and DPP-IV inhibitor. Participants are asked to fast overnight before clinic visit next morning. At clinic a fasting blood sample is taken, and participants then consume a breakfast that is consistent with their diet regimen i.e., a meal that is standard for them, not an experimental-defined standard (or a light beverage e.g., coffee, for those participants whose diet does not include breakfast meal). Three further post-prandial blood samples are taken for analysis.

° One pre-prandial timepoint and 3 post-prandial (30 minutes, 1 hour, 2 hours).

- Participants' physiological stress via pre-meal salivary cortisol—collected by non-invasive passive drool.

° Variability in cortisol concentrations are higher and expected in morning after waking or eating prior to sampling (77)—participants will be prompted with additional pre-collection questions (e.g., “Has this been a normal morning for you?”)

- Gut microbiome analyses—collection of feces/stool by a self-collection kit (OMNIgene-GUT, DNA Genotek) at four time points.

° Baseline and 1-month follow-up will require one sample taken within 24–48 h of clinic visit.
° Final 3-month follow-up clinic visit requires two independent samples: one within 24–48 h of clinic visit and the second between 3 and 7 days.

- Exercise, movement and sleep actigraphy—at the end of their first in-person session, participants will be provided with a Fitbit, to be worn for the duration of the study.

### 2.4 Presentation of outcomes

Part 1

- Diet motivation categorization: a measure of the frequency and distribution of motivations across the participants.
- Diet Compliance/Adherence: a composite score generated through factor analysis of collected items related to whether participants adhered to their intended diet length, self-reported following their original diet plan and the consistency of food intake and diet-related activities with original diet plan.
- Diet Success: an idiographic variable determined by the achievement of intended diet goals (e.g., obtaining weight goal, improved physical health).

Part 2

- Neuroendocrine State Measures: The heterogeneity of neuroendocrine markers in the study population at baseline (“pre-diet”) will be assessed. Potential changes in neuroendocrine state within individuals associated with dieting will be determined by comparison between pre-diet and on-diet measures.

° Determination of fasted-state and post-prandial response for selected peptide hormones in blood.

▪ Amylin, Cholecystokinin, FGF-21, GDF-11, GDF-15, Ghrelin, GIP, GLP-1, Glucagon, Insulin, Leptin, Pancreatic Polypeptide, Peptide Tyrosine Tyrosine, Secretin

° Selected cytokines as immunological markers

▪ IL-6, TNFα

° Stress hormones

▪ Cortisol concentration in saliva

- Physical Change associated with diet: BMI, Blood Pressure, Defecations/Bowel Movements, Sleep
- Microbiome state measures: microbiome will be analyzed from 16S amplicon sequence datasets. Community structure described via alpha-(Chao, Shannon indices) and beta-diversity metrics.

° The heterogeneity of gut microbial communities across the study population will be addressed using baseline stool samples.
° Dynamics of individuals microbiomes over time will be assessed using their longitudinal series of 4 samples.

### 2.5 Data analysis plan

#### 2.5.1 Parts 1 and 2

Our broad research question relates to the impact and interplay of psychological and biological mechanisms which may influence food intake behaviors, adherence, diet success and outcomes. As the first in-depth study of self-directed dieting in young people, we also aim to detail the behaviors and characteristics of young people on a diet, using descriptive statistics. All participant data will be de-identified during analyses. Data will be exported from Teamscope, University of Sydney's licensed REDCap and Fitbit for statistical analysis. The widely used diet and nutritional analysis program, FoodWorks 10 Professional will be utilized to analyse dietary food intakes. Combined psychological, biological and physiological data will be processed in programs STATA, R studio and GraphPad Prism utilized for powerful biostatistics, curve-fitting, and scientific-graphing tools. Initial exploratory analysis will be conducted with descriptive statistics to summarize the results with measures of central tendency and dispersion. Factor analysis will be explored and used to reduce dimensionality where it is required and appropriate prior to further analysis and modeling. Greater than 5% of missing data will be considered significant and analyzed further with appropriate statistical models. This will include multiple imputations and generalized linear mixed modeling (GLMM) based on missingness assumptions that fit the data (missing at random vs. missing not at random) and the most appropriate method for analysis with intention to treat principles.

#### 2.5.2 Part 2

Our research questions in this part of the study are broadly to characterize the range of variation in selected aspects of the gut-brain axis, predicted to be susceptible to change in response to dieting. We expect the greatest change from diet components to be in circulating metabolites, found through in-person clinic collected blood samples. Systems biology and computational workflow in R studio will be used to process biological data. Metabolomics will be assessed from overall magnitude of measurable response, peak concentrations and change pre- and post-prandially. These blood samples will also be used to assess peptide hormones and cytokines via Enzyme-linked immunosorbent assay (ELISA). Targeted analytes include appetite, gut and metabolic hormones such as Fibroblast Growth Factor 21 (FGF-21) and cytokines such as Interlukin-6 (IL-6) to assess fasted state and post-prandial responses. Fasted state saliva samples will be used to assess cortisol, and also stored for potential genetic analyses.

Fecal samples will be used to extract metagenomic DNA and assess microbiome state via 16S amplicon sequencing. Gut microbiome analyses will be based on an initial classification of the data sets to amplicon sequence variants (ASVs) and assignation of these to current taxonomic references. Analyses of alpha and beta diversity patterns and association with psychometric and physiological data sets will be recapitulated at multiple taxonomic levels from the finest scale (ASV) to higher taxa (78). We will use ASV taxonomic assignations to reconstruct predicted genome traits of taxa of interest (79–82). To account for dynamics in cell density and alpha-diversity indices, variations within an individual's stool (e.g., a watery sample) compared to a diet (83), will be compared to 4-day stool diaries.

### 2.6 Interpretive context—Limitations and strengths

To the best of our knowledge, no longitudinal study with repeated measures across time has investigated the association between diet maintenance and compliance in conjunction with physiological sampling. A feature of the current study is that participants may perceive their foods in different ways, and engage with daily recording at different times and therefore record their online food logs and portions inconsistently. A consequence of the this is high levels of variance in foods consumed as part of the self-driven diets, especially for Part 2 participants giving biological samples. However, this is also strength as it emulates realistic dieting practices. Another strength overall to the study is that we are recruiting a large sample in Part 1 as a representative of the community, and provide frequent check-ins to assess diet change and drive retention.

## 3 Discussion

The My Diet Study will be the first in-depth study of psychological and biological processes in the natural dieting process in a cohort that report very high rates of self-directed dieting—young people. The study aims to examine the underlying psychological, behavioral and physiological mechanisms which influence feeding behavior and the associated physical and mental outcomes of naturalistic dieting among young people. It is hoped that this will help identify biomarkers and early indicators of adherence to self-directed goals, compliance and outcomes including risk of developing an eating disorder, for early intervention.

Within our target cohort of adolescents and young adults (*N* = 500), it is expected we will be able to track natural variation in dieting compliance. We hope to establish a community-based longitudinal dataset on young dieters with information on individuals' physical and mental health, dieting adherence and perceived success. Findings are expected to help identify specific diets or eating behaviors which may be associated with negative outcomes, such as greater depressive symptoms, extreme weight control behaviors (e.g., fasting, purging) and non-compliance with dietary guidelines for healthy eating.

For ethical and safety reasons, individuals who screen at risk of eating disorder or self-report a current eating disorder will be excluded prior to entry. Previous studies have found self-reported dieting to be a risk factor for eating disorder development over time (52, 84–86). Further, in studies on the etiology of eating disorders, dieting is a precursor to eating disorder development (85–87). Consistent with a ethical “do no harm” approach, participants with increasing scores on eating measures given throughout the study will also be monitored by research team members with clincial expertise expertise in eating disorders, and any participant who meets threshold criteria for an eating disorder (55) at any point in will be excluded from the study and referred on to adequate support services. Genetic information collected from a subsample of participants (*n* = 50) will also be analyzed for polygenic risk scores for anorexia nervosa, bulimia nervosa and binge-eating disorder, to determine whether any dieters may have a greater genetic propensity for eating disorder development. Prior research has identified a number of loci and genes implicated in anorexia nervosa (88).

To date, much of the research has focused on dieting as largely a conscious behavior, determined by individual motivation and other cognitive processes (89), ignoring the influence of our underlying physiology in determining eating behavior. The My Diet Study, a naturalistic, observational longitudinal study seeks to address this gap in the literature by examining psycho-biological processes in dieting adherence, collecting a range of physiological and biological markers from young people on a self-directed diet. Findings may shed light on potential biopsychosocial solutions (e.g., psychoeducation around the nutritional value of foods which contain good bacteria) for improving diet adherence with hopes to identify early markers of healthy and unhealthy dieting to inform future interventions.

## Ethics statement

This study has been approved by the Human Ethics Review Committee (RPAH Zone) of the Sydney Local Health District (X21-0181). This study will be conducted in accordance with the *National Statement on Ethical Conduct in Human Research* (2007), the *CPMP/ICH Note for Guidance on Good Clinical Practice*, and consistent with the principles that have their origin in the Declaration of Helsinki. Compliance with these standards provides assurance that the rights, safety and wellbeing of trial participants are respected. Participants will be required to give online informed consent prior to completing any study measures.

## Author contributions

MO: Conceptualization, Funding acquisition, Investigation, Visualization, Writing—original draft, Writing—review & editing. MP: Investigation, Visualization, Writing—original draft, Writing—review & editing. JM-W: Conceptualization, Writing—review & editing. SB: Investigation, Writing—review & editing. KG: Supervision, Writing—review & editing. ST: Supervision, Writing—review & editing. SS: Conceptualization, Funding acquisition, Resources, Supervision, Writing—review & editing. SM: Conceptualization, Funding acquisition, Resources, Supervision, Writing—review & editing. AH: Conceptualization, Funding acquisition, Resources, Supervision, Writing—review & editing.

## Footnotes

^1^ https://www.mydietstudy.com.au/
