# Supplementary material for: My Diet Study: protocol for a two-part observational, longitudinal, psycho-biological study of dieting in Australian youth
Source: Front Public Health. 2023 Dec 14;11:1281855. doi: 10.3389/fpubh.2023.1281855 (PMC10752999; doi:10.3389/fpubh.2023.1281855)
Supplement: Supplementary file 1 [file Table_1.docx]

**Baseline Questionnaire**

Have you started your diet yet? (Yes/No)

When do you intend to start your diet?

1. Immediately

2. In the next month

3. In more than a month’s time

What is your date of birth? (age automatically calculated based on date)

What is your sex assigned at birth?

Female

1. Male

Which of the following describes your current gender identity?

1. Female

2. Male

3. Non-binary/Gender fluid

4. Different identity: (free text)

5. I prefer not to say

6. Not listed

7. Please specify gender not listed (free text)

What is your postcode?

Do you have a clinically diagnosed gastrointestinal disorder, formal diagnosis of malnourishment, or major medical condition that requires you to comply with a particular diet? (Yes/No)

Are you currently pregnant or lactating? (Yes/No)

Has a medical doctor prescribed you this diet? (Yes/No)

How long do you intend to be on your diet? (0 months – 6+ months)

Have you ever received any of the following eating disorder diagnoses?

[For all: Currently (active) – Previously (recovered) – I have had symptoms but no formal diagnosis – No]

1. Anorexia Nervosa
2. Bulimia Nervosa
3. Binge Eating Disorder
4. Avoidant/Restrictive Food Intake Disorder
5. Other or Unspecified Feeding / Eating Disorder  (e.g., Atypical AN, Purging Disorder)
6. Unsure of specific diagnosis

Please indicate if you have ever engaged in the following behaviours:

[For all: Currently – Previously – I don’t know – No]

1. Food restriction/dieting
2. Over/binge eating
3. Self-induced vomiting
4. Driven over exercise
5. Laxative and/or diuretic misuse
6. Diet pill misuse
7. Body image concern
8. Other (free text)

Are you a University of Sydney Psychology student participating in this study in return for partial course credit? (Yes/No)

**Demographics:**

What is your ethnicity? ****optional****

1. Australian
2. Indigenous Australian or Torres Strait Islander
3. New Zealander
4. Asian
5. Indian
6. Middle Eastern
7. European
8. North American
9. South American
10. African
11. Other, please specify

What is your religion? ****optional****

1. Christianity
2. Islam
3. Buddhism
4. Hinduism
5. Judaism
6. No religion
7. Other, please specify

What is your current work status?

1. Full time paid work
2. Part time paid work
3. Unemployed and looking for work
4. Unemployed and not looking for work

Are you currently studying?

1. Yes, high school
2. Yes, trade certificate, diploma, or apprenticeship
3. Yes, university undergraduate degree
4. Yes, university postgraduate degree
5. No

What is the highest level of education that you have ever completed?

1. Primary school
2. High school
3. Trade certificate, diploma, or apprenticeship
4. University undergraduate degree
5. University postgraduate qualification
6. Other (free text)

What is your current living situation?

1. Alone
2. With parents
3. With friends
4. In a share house/student accommodation
5. With partner (no kids)
6. With partner (with kids)
7. Other (free text)

**Current diet:**

Have you started your diet yet? (Yes/No)

When do you intend to start your diet? Please tell us in how many weeks or days? If possible, please specify the exact date. (e.g. In 2 weeks, 24 January 2021) OR (In 10 days, 24/01/2021)

Can you please describe what your plan is for your diet? (eg. eating paleo, switching to a keto diet for first month and then to a low-carb diet after) (free text)

How did you find out about this diet?

- I did my own research via google search, on websites
- through an email
- was recommended by a friend
- was recommended by a health professional
- Other, please specify

What is your goal of this diet? (eg. to gain muscle mass, to lose weight) (free text)

How confident are you that you will reach your goal? (not confident – very confident)

Do you have a particular weight goal? (Yes-No)

What’s your weight goal? (Please describe this in kg) (numerical)

How confident are you that you will stick to your diet plan? (not confident – very confident)

What other activities will you engage in while you are on this diet (e.g., exercise, using an app/program, etc)? (free text)

Have you been, or do you plan to, drink protein powder, collagen, diet drinks? Please describe. (free text)

Have you been, or do you plan to, take any supplements, vitamins, laxatives or pills to supplement the diet? Please describe. (free text)

Have you been, or do you plan to, take any prescribed medications for the diet? Please describe. (free text)

**Diet History:**

Have you tried any of the following diets? (Matrix including asking when they used the diet, for how long did they use the diet, the goal of the diet, and to rate 1-5 if the diet achieved their goal)

1. Atkins Diet
2. Vegetarian
3. Pescatarian
4. Vegan
5. Paleo
6. Keto
7. Gluten-Free
8. Mediterranean diet
9. Carnivore diet
10. Intermittent fasting
11. Halal
12. Kosher
13. Meal Replacement diet
14. Clean Eating diet
15. Juice Cleanse diet
16. Flexitarian Diet
17. Zone diet
18. Raw diet
19. Dubrow diet
20. Dukan diet (high-protein, low-carb)
21. No-sugar diet
22. Nordic diet
23. Low-carb diet (20-150g per day)
24. Carb cycling diet
25. Ultra-low-fat-diet
26. DASH diet
27. MIND diet (DASH x Mediterranean)
28. FODMAP
29. CSIRO Total Wellbeing Diet
30. Volumetrics diet
31. Other (free text)

Has the reason for your dieting historically been related to any medical conditions? (free text)

Has the reason for your dieting historically been related to any religious reasons? (free text)

**Health status:**

How is your physical health in general? (Very good – very poor)

Do you have any underlying health conditions? (Free text or yes/no)

In the last month, have you had any treatment of antibiotics or steroids? (Yes/No)

Do you faint / feel faint easily? (Yes/No)

Do you have any diagnosed mental health concerns? Yes (please specify)/No

During the past month, what time do you normally wake up? (numerical text)

During the past month, how would you rate your sleep quality overall?

[Very Good – Fairly Good – Fairly Bad – Very Bad]

During the past month, when have you usually gone to bed at night? (free text)

During the past month, how long (in minutes) has it usually taken you to fall asleep each night? (numerical text)

During the past month, how many hours of actual sleep did you get at night? (This may be different than the number of hours you spend in bed) (numerical text)

When you hear about “morning types” and “evening types”, which one of these types do you consider yourself to be?

1. Definitely a morning type
2. Rather more a morning type than an evening type
3. Rather more an evening type than a morning type
4. Definitely an evening type

**Family Health Background:**

Has anyone in your family had an experience of an eating disorder before? Yes (please specify)/No

Do you have a family history of obesity at all? Yes (please specify)/No

**Follow-Up Questionnaires**

**Current diet:**

In the past month, overall, how has your diet been going? Have you continued, changed or ceased your diet?

- Continued as per original plan
- plan has changed
- I am going on and off the diet
- I have stopped dieting

In the past month, have you stuck to your original diet plan? (Not at all – Very much so)

If it has changed, how has your diet plan changed? (free text)

If you are still dieting, how confident are you that you will stick to your diet plan in the next month? (not confident – very confident)

If you are still dieting, how long do you intend to be on this diet? (0 months – 6+ months)

If you have ceased dieting, do you think you will return to the diet in the next month? (Not likely – Likely)

Have your dieting goals changed? (free text)

How confident are you that you will reach your goal? (not confident – very confident)

In the past month, what other activities have you engaged in (e.g., exercise, food diary keeping, etc)? (free text)

In the past month, have you been drinking protein powder, collagen, diet drinks? Please describe. (free text)

In the past month, have you been taking any supplements, vitamins, laxatives or pills? Please describe. (free text)

In the past month, have you been taking any prescribed medications for the diet? Please describe. (free text)

How stressful do you find it trying to stick to this diet? (Not stressful, A bit stressful, Feel very stressed trying to stick to this diet)

**Health status:**

How is your physical health in general? (Very good – very poor)

In the past month, have you had any illnesses or new medical diagnoses? (Yes (please specify)/No)

In the past month, have you had any treatment of antibiotics or steroids? (Yes/No)
