# Supplementary material for: My Diet Study: protocol for a two-part observational, longitudinal, psycho-biological study of dieting in Australian youth
Source: Front Public Health. 2023 Dec 14;11:1281855. doi: 10.3389/fpubh.2023.1281855 (PMC10752999; doi:10.3389/fpubh.2023.1281855)
Supplement: Supplementary file 2 [file Table_2.docx]

**Food and Mood Diary**

Date & Time of Meal: [DD-MM-YYYY, Time HH:MM AM / PM]

Meal – [Breakfast, Lunch, Dinner, Dessert, Snack]

- *Breakfast/Lunch Selected:* What time did you go to bed last night? (time HH:MM AM/PM)
- *Breakfast/Lunch Selected:* How would you rate your sleep quality overall? (Very good, fairly good, fairly bad, Very bad)
- *Breakfast/Lunch Selected:* What time did you wake up today? (time HH:MM AM/PM)

Did you skip this meal? [Yes/No]

Can you please describe what you are eating – (I.e., a brief description of the type of food, include brand details if known and the quantity/how much food there is. If you do not have measuring cups or scales at home or are eating out, describe the food you eat using household measures)

[e.g. 1 medium slice Dominos pizza; 1 cup of veg and 200g beef rump steak, large glass of lemonade]

Did you consume any alcohol with this meal? - [No, 1 standard drink, 2-3 standard drinks, 4+ standard drinks]

- *Alcohol option selected:* Please describe your alcoholic drink (eg. 1 standard 350ml of Corona beer)

How was your meal prepared? [I prepared/cooked it, Someone else home-prepared/cooked it for me (e.g., Family member), I bought it from a grocery store (e.g., ALDI, Coles, Woolies), I bought from a café/restaurant, Bought fast food (e.g., McDonalds), Other (Please describe other)]

Where are you eating? – [Home, work, school/study, out socially (eg. at a café), other (Please describe other location)]

Is anyone with you? – [Family, friends, Eating alone out in public, No , other (Please describe other eating situation)]

Is this meal part of your diet plan – [Yes / No]

Which of the following best describes how you feel about this meal? [I didn’t eat enough, Just enough, I overate]

Did you want to eat because you were feeling hungry? [Yes/ No]

How motivated are you at the moment to stick to a diet – [1, Not at all – 4, Very much so]

These next questions are after you finish your meal

How anxious do you feel after eating? [Calm/Relaxed – Anxious]

How is your mood right now after eating – [Happy – Sad]

How guilty do you feel after eating? [Not guilty – Guilty]

Other mood: Please specify [free text]

How hungry are you right now – [1, Not hungry at all – 5, Very hungry]

Thank you, hope you enjoyed your meal!

**Stool Diary**

Please complete the following reporting symptoms with each bowel motion (stool/defecation) today.


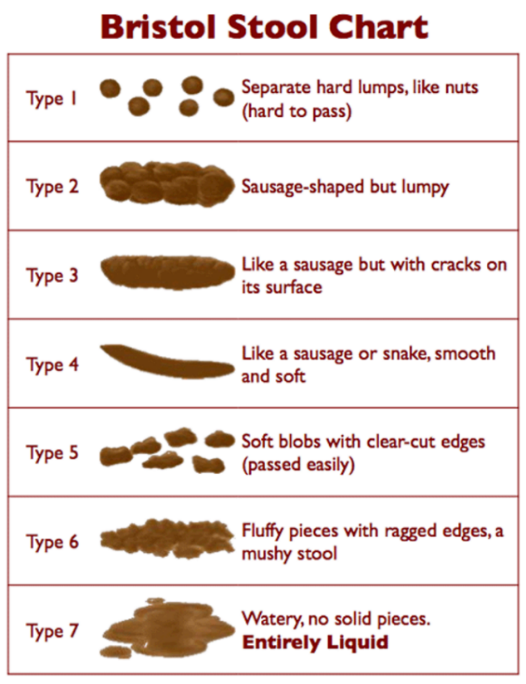


**Bowel movements - Day 1**

**I didn’t poo TODAY :(** *No poop* ☐

1. **Approximate time:** *(select one)*

- Morning (eg. As you’ve woken up, before leaving home)
- During Daily Routine (eg. At lunch, at work, outside of home)
- Evening (eg. 6pm – midnight, before bedtime)
- Woke up from sleep to go

1. **What did your faeces look like – Use the Bristol stool chart supplied** *(please select the number)*
2. **Were there any distinctive aspects of your faeces?**

**(e.g. Mucus, blood, pain, colour, etc)**

|  | | **(1) Not at all** | **(2) Slightly** | **(3) Moderately** | **(4) Significantly** | **(5) Unable to control / had an accident** |
| --- | --- | --- | --- | --- | --- | --- |
| **4** | **How much did you strain?** |  |  |  |  |  |
| **5** | **Abdominal pain before poo?** |  |  |  |  |  |
| **6** | **Did you feel the urge to go?** |  |  |  |  |  |

|  | | **Yes** | **No** | **Not Applicable** |
| --- | --- | --- | --- | --- |
| **7** | **Abdominal pain relieved by bowel motion?** |  |  |  |
| **8** | **Abdominal pain worsened by bowel motion?** |  |  |  |
| **9** | **Use of laxatives?** |  |  |  |

**10. Is this the stool/poo you’re bringing in for analysis?** (Yes / Not Applicable)

|  | | **(1) Never** | **(2) A little**  (eg. only once or twice) | **(3) Moderately**  (eg. every 3-4 hours) | **(4) A lot**  (eg. Every 2hours, every hour) |
| --- | --- | --- | --- | --- | --- |
| **11** | **How often did you fart today?** |  |  |  |  |

**12. Were they smelly?** (Yes / No) **Not prompted if ‘Never’ selected*

Fun fact: Any odour means amines or sulfate compounds are present and just means your gut microbes are working differently! If there’s no smell, there’s still gas present but just different types.
